# Effect of Physical Activity on Symptoms of Morphine Addiction in Rats, after and before of Lesion of the mPFC Area 

**Published:** 2013-10

**Authors:** Vajiheh Saedi Marghmaleki, Hojjat Allah Alaei, Hamid Azizi Malekabadi, Aliasghar Pilehvarian

**Affiliations:** 1 Department of Basic Sciences, Isfahan Payam -e -Noor University, Isfahan, Iran; 2 Department of Physiology, School of Medicine, Isfahan University of Medical Sciences, Isfahan, Iran; 3 Department of Basic Sciences, Islamic Azad University, Khorasgan Branch, Isfahan, Iran; 4 Department of Basic Sciences, Isfahan Payam-e- Noor University, Isfahan, Iran

**Keywords:** *Exercise*, *Morphine*, *mPFC*, *Withdrawal symptom*

## Abstract

***Objective(s):*** Drug addiction is one of the fastest growing social problems that researchers, for many years, have been trying to find proper strategies for its prevention and treatment. Short-term physical activity is considered as acceptable for the treatment and prevention of addiction. On the other hand, prefrontal cortex is known to be responsible for many of the complex behavioral responses, especially those relevant to addiction and drug abuse. In this study, the effects of short-term physical activity (treadmill running) on withdrawal symptoms in rats, with or without medial Prefrontal Cortex (mPFC) area was evaluated.

*** Materials and Methods:*** In this experimental study, male Wistar rats, weighing 250-300 g. Were selected and divided into four groups: control, sham, test1 and test 2 groups, with one group having a surgery (test 2). In order to study the effects of short-term physical activity, we employed a treadmill with the adjustable speed of 17 m/min, and a 15% incline. Following the injection of three different doses of morphine (10, 20 and 40 mg/Kg) for 9 days, treadmill running was completed on the tenth day and then the symptoms of addiction were evaluated.

***Results:*** Administration of morphine as aforementioned, and treadmill running on rats with mPFC area, significantly alleviated some of the symptoms and signs, such as, bruxism, cycling, body lift, licking, standing and scratching (*P*<0.05). Therefore, there are noticeable differences in these symptoms between the exercise and morphine groups (*P*<0.05).

***Conclusion*** *:* We propose that when using morphine mPFC plays an important role in the reduction of reward symptoms. Running lessens the effects of morphine. Moreover, short-term physical activity might decrease the tendency to use morphine in those with mPFC area, compared to other groups, especially with the one undergoing an mPFC operation.

## Introduction

Researchers described addiction as a disease which changes the molecular and physiological factors which cause addiction to morphine (1). Several studies have shown the effective role of therapeutic exercise in treatment and even prevention of many diseases, from depression to memory loss in Alzheimer's disease (2, 3). Most of these studies have demonstrated that different aerobic exercises are associated with similar findings (2, 4, 5). These findings suggest that exercise has certain effects on the brain activity, by mechanisms which increase the release of various neurotransmitters (4-6).

Therefore, we may consider exercise as a valuable factor in the treatment of addiction to drugs (7). On the other hand, it is believed that the endogenous opioid system is effectual in learning and memory acquisition (8).

Opioid receptor agonists, with a certain dose and duration, when injected after training, have a detrimental effect on memory (9). The damaging effects of the opioid receptor antagonists, such as naloxone are partially due to the involvement of opioid receptors in morphine state-dependent learning (STD). 

However, pharmacological findings suggest that the same neurotrophic factors modulate learning, memory and drug addiction. Also the same brain regions are considered important sites for the molecular and cellular plasticity in drug addiction and memory. 

Complex circuits involving the hippocampus, cerebral cortex, ventral and dorsal striatum, and amygdala are implicated in both addiction and memory (1). Human and animal studies have suggested that exercise also has beneficial effects on brain functions, including the promotion of plasticity, the enhancement of learning and memory and the prevention of addiction. Dopamine is one of the neurotransmitters that has an important role in the improvement of memory and development of addiction. A dopaminergic pathway, extending from the brain stem to the forebrain, is involved in the reward/reinforcement system (10). Both exercise and drug addiction change the level of some neurotransmitters, such as norepinephrine and glutamate which reduce in the bed nucleus in chronic morphine-treated rats (11). Addiction to some drugs such as cocaine and amphetamine, act directly on the dopaminergic system. While, heroin and alcohol indirectly influence the dopaminergic neurons, which seem to be the mediators of addiction to cocaine (12).

Recent experimental and clinical findings suggest that physicians can use exercise to treat patients with addiction disorders, since the prolonged rhythmic exercise may activate the central opioid systems. It could be an important step toward the development of new treatment modalities for addiction, and leading us toward an association of exercise with addiction, learning and memory (13). 

Prefrontal cortex has strong interactions with the thalamus, hippocampus, cerebral cortex and amygdale, along with many complex behavioral responses, such as the reward-oriented behavior, which is observed in those involved in drug abuse and addiction. On this account, prefrontal cortex, according to the receiving nerves, is divided into three parts including: medulla prefrontal cortex or mPFC, lateral prefrontal cortex (lPFC) and a middle area. Based on what was mentioned above, mPFC is implicated in addiction to morphine. 

Considering the anatomical and functional relationship between mPFC area and the withdrawal symptoms of addiction, in this study we investigated the effects of short-term physical activity on withdrawal symptoms, after the administration of morphine in male rats, either having or lacking mPFC area.

## Materials and Methods

***Experimental animals***

This experimental study was performed on 40 male Wistar rats, weighing approximately 250-300 g. The male rats were selected to avoid the probable effects of estrous cycle on test results. The Ethical Committee for Animal Experiments in Isfahan University of Medical Sciences approved the study protocol and all experiments were conducted in accordance with the international principles for biomedical research involving animals, revised in 1985.

The rats were housed in 4-5 groups in cages, and they were kept under controlled conditions (temperature 20-24°C, relative humidity 40 to 70 % and light/darkness cycle 12/12 hr (lights on at 8:00 a.m.). Food and water were available *ad libitum*. The measurements were constantly made during the first half of the light cycle. Rats were pre-tested to determine their treadmill-running willingness and those which refused to run, were excluded before the experiments started.

Rats were divided into four groups as follows: 

1) Control group (exercise and saline group) which received intra-peritoneal (IP) 0.2 ml saline (9% NaCl) and participated in treadmill exercise sessions (1 hr at the speed of 17 m/min and with an incline of 15%) for 10 days.

2) Sham group (exercise, morphine and surgery group), which initially underwent a stereotaxic operation. And then received intra-peritoneal morphine as follows: first 3 days 10 mg/kg, next 3 days 20 mg/kg, and during the last 3 days 40 mg/kg. Meanwhile, after doing the exercise, the rats received a dose of morphine every day. (See control group).On the tenth day, the symptoms of addiction were evaluated. 

3) Test-one group (morphine group), which received intra-peritoneal morphine as follows: first 3 days 10 mg/kg, next 3 days 20 mg/kg and during the last 3 days, 40 mg/kg. On the tenth day, the symptoms of addiction were evaluated. 

4) Test-two group (exercise and morphine group), in which each morphine dose (see group 3) was preceded by exercise (see group 1). On the tenth day, the symptoms of addiction were evaluated. 

***Behavioral apparatus and method***

*Exercise*

In order to investigate the effects of short-term physical exercise, we tested running on a treadmill with a rectangular metal frame and 24 × 43 × 245 cm dimensions. Each rat ran at certain times of day. In fact, this method provides the opportunity to remove the sick or lazy rats from the groups. To minimize the stress, rats were familiarized with the treadmill running as described above. All these animals tolerated the speed (17 m/min), the incline (15 %) and the duration of exercise, and successfully completed the training program.

*Stereotaxic surgery*

To perform surgery, each rat was initially anaesthetized with intraperitoneal chlorohydrate (0.5 mg/100 ml) and the animal’s head was fixed in a stereotaxic apparatus [Manufacturing Co. Steoling USA]. After cleaning the target point on the surface of the skull and determining the Bregma and Lambda areas using an atlas (Paxinos Atlas) (14) as the coordinate reference of the mPFC area (AP =3.2, DV = 2.5 and L = 0.6), the mPFC nucleus was destroyed. Finally, the rats were transferred to separate cages under full care. After three days they were used for proceeding with the project. 

*Withdrawal syndrome signs*

The withdrawal syndrome was precipitated with an intra-peritoneal injection of 2.0 mg/kg naloxone HCl, dissolved in saline. We recorded the withdrawal signs within 30 min after the injection of naloxone on the previous day. Abstinence signs were advanced by naloxone in three experimental groups, mainly including: cycling, standing, scratching, licking, body lift, scratching and bruxism.

*Data analysis*

Data analyses were carried out by means of ANOVA, with group as the independent variable, and performance in each session, as the dependent variable. Mixed ANOVA analyses were performed with their corresponding contrast analyses. All the data were expressed as mean±SEM (*P* <0.05). The data of different groups were compared using ANOVA test with Turkey's *post-hoc*.

## Results

The survey results demonstrated that exercise has a detrimental effect on tendency to use morphine. The rats’ exposure to morphine and exercise had particularly affected their memory and learning. Significant differences were observed between control and test-one groups that only received morphine.

**Figure 1:**
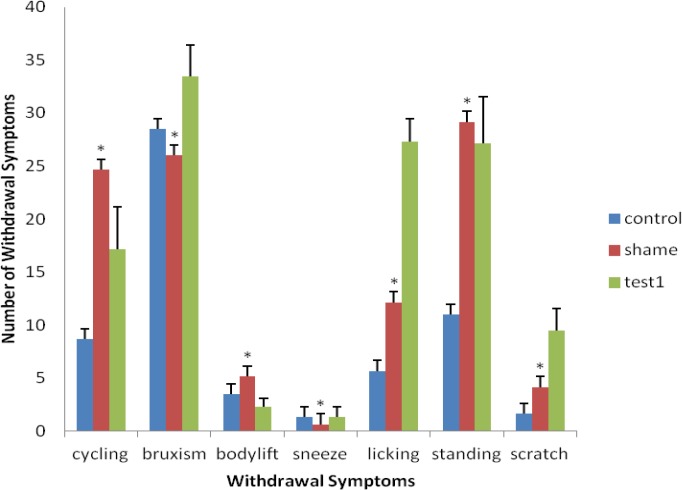
Comparison of withdrawal symptoms in test 1 with the sham group (*significant difference with test 1; the results are shown as mean ± SEM,) demonstrates that no significant difference is present among the groups (*P*> 0.05)

Although, exercise was effective on withdrawal symptoms and reduced the signs, according to the statistical analysis, damage to mPFC area, which causes this reduction, did not show a significant difference (*P*> 0.05) (Figure 1). 

**Figure 2:**
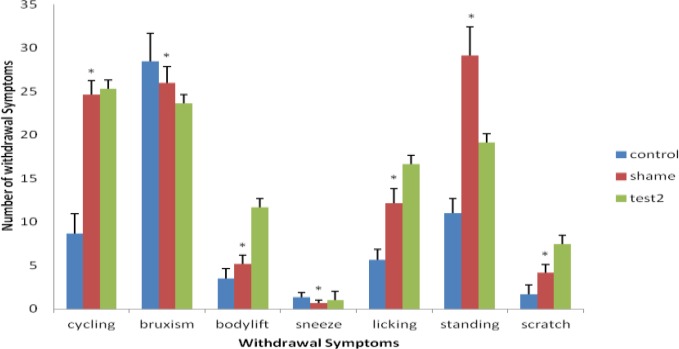
Comparison of withdrawal symptoms in test-2 with the sham group (* significant difference with test 2; results are shown as mean ± SEM and (*P* <0.05). A significant difference is present among the groups

On the other hand, comparison between the sham and test-two groups, which were treated with morphine and exercise for ten days, indicated certain differences (*P*<0.05).This means, the rats, treated with morphine and exercise, were able to relieve the withdrawal symptoms better than the sham group, which after having sterotaxic surgery were treated with morphine and exercise. Therefore, mPFC area and exercise had a certain effect on weakening the tendency to use morphine (Figure 2). Hence, mPFC area has a significant effect on learning, memory and addiction to opioid drugs.

**Figure 3:**
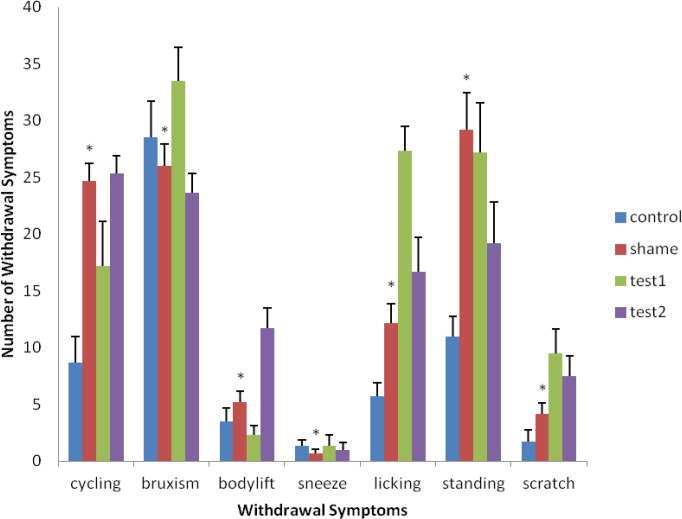
Comparison of the effects of withdrawal symptoms in the exercise group, having morphine and surgery, with the morphine group. This chart shows the reduction of tendency to use morphine in rats which were treated with surgery, morphine and exercise compared with the morphine group (* Significant differences with test groups). [Results are shown as mean ± SEM and (*P* <0.05)]. A significant difference is present among the groups

## Discussion

The maintenance of brain health and plasticity is an important public health ambition. It is understood that physical exercise can help people remain healthier (15).

 Our findings emphasize the role of physical activity, and probably mPFC area activities, in the promotion of learning and memory functions. The studies in this paper indicated that exercise decreases the tendency to use morphine and treats the rats receiving morphine. Moreover, they revealed that exercise has a major effect on learning and memory, and this issue was particularly demonstrated in the comparison with the sham group (7, 16, 17).

 According to these results and other studies, the main cause of the positive interaction of morphine with exercise and neural mechanisms is the similarity between the roles of various responsible brain areas, while mPFC area was not yet clarified (22-24). 

 Previous studies have shown the release of endogenous opioids following exercise (25) and that these endorphins increase the feeling of pleasure during workouts (26). Moreover, the evidence has proven that exercise has encouraged the affected rats, and this perhaps was mediated by the opioid system (27).

 Literature review suggests that the levels of endogenous opioids are increased by exercise. One study has demonstrated that addiction can be considered an endorphin deficiency disorder (28). Possibly, one reason for the weakening tendency to use morphine after exercise, as shown in the present study, is the activation of the endogenous opioid system. In addition, exercise influences the central dopaminergic and glutamatergic systems (5).   The importance of dopaminergic system in morphine dependency and in response to reward/reinforcement system, is in the relevance of physical activity and drug abuse, as determined by several studies (29, 30). 

In another experiment, it has been reported that exercise changes the level of dopamine in the brain (3) and its metabolism increases in certain regions of the brain during workouts (3). Furthermore, as shown in this study, it seems that exercise interferes with dopamenargenic system and changes the level of dopamine in different parts of the brain, especially in mPFC area. Subsequently, many efforts have been made to determine the role of mPFC area in cognitive performances (28). 

 MPFC area is composed of four main sections: The dorsal side to the abdomen part, the middle granular region, the superior belt region, pre-limbic cortex and inferior cortex. There are many different kinds of neurons in mPFC area such as glutamate inhibitory cells, cholinergic efferent, medial cholinergic and GABAergic neurons. The efferent glutamatergic neurons from mPFC area enter VTA and Accumbens areas. Anatomical correlation between mPFC nucleus and accumbens and ventral tegmental area (VTA) shows that mPFC area has a strong role in this system. Therefore, activities of the mesolimbic system have important effects on reward responding (10). Destruction or inactivation of mPFC area decreases the reward responding with other mechanisms. As a result, exercise has certain effects on releasing the endorphins and activating the neurotransmitter pathways such as, serotonergic and glutamatergic systems (20, 29-31). 

Shortly, the effect of short-term physical activity on withdrawal symptoms, with or without mPFC nucleus, in morphine-dependent rats was investigated. 

## Conclusion

As shown in the present study, it is likely that short-term exercise, by activating the opioid system and with the activity of mPFC area, increases learning and memory in the addicted rats. Additionally, it decreases these animals’ tendency to use morphine.

Therefore, what remains to test is the effect of medium- and long-term exercise on the endorphin release and on other neurotransmitters in the brain.

## References

[1] Nestle EJ (2002). Neurobiology Learn. Common molecular and cellular substrates of addiction and memory. Neurobiol Learn Mem.

[2] Kimmie Mc (2005). Marnie Walk away from depression.

[3] Meeusen R, Piacentini De Meirleir K (2001). Brain microdialysis in exercise research. Sports Med.

[4] Azizi Malekabadi H, Alaei H, Oryan S (2007). The effect of exercise (treadmill running) on glutamate concentration variation of hippocampal dentate gyrus in the intact and morphine dependent male rats. Iran J Basic Med Sci.

[5] Bequet F, Gomez- Merino D, Bertheiot M, Guezennec CY (2001). Exercise-induced changes in brain glucose supplementation and serotonin revealed by microdialysis in rat hippocampus. Acta Physiol Scand.

[6] Meeusen R, Piacentini MF, Van Den ES, Magnus L, De MK (2001). Exercise performance is not influenced by a 5-HT reuptake inhibitor. Int J Sports Med.

[7] Azizi Malekabadi H, Alaei H, Hsseini M (2008). The effect of short-term physical activity(treadmill running) on spatial learning and memory in the intact and morphine dependent male rats. J Isfahan Med School.

[8] Kavoosi Z, Fereidoni M, Moghimi A, SadeghA (2009). Effect of administration of ultra low doses of morphine on learning processes and spatial memory retention in morris water maze in rat..

[9] Poulsen FR, Meyar M, Rasmussen JZ (2003). Generation of new nerve cells in the adult human brain. Ugeskr Laeger.

[10] Berke JD, Hyman SE (2000). Addiction, dopamine, and the molecular mechanisms of memory. Neuron.

[11] Fuentealba JA, Forray MI, Gysling K (2000). Chronic morphine treatment and withdrawal increase extracellular levels of norepinephrine in the rat bed nucleus of the stria terminalis. J Neurochem.

[12] Berke JD, Hyman SE (2000). Addiction, dopamine, and the molecular mechanisms of memory. Neuron.

[13] Alaei H, Borjeian L, Azizi M, Orian S, Pourshanazari A, Hanninen O (2006). Treadmill running reverses retention deficit induced by morphine. Eur J Pharmacol.

[14] Paxinos G, Watson C (2006). The rat brain in streotaxic coordinates.

[15] Koyuncuoglu H, Nurten A, Enginar N, Ozerman B, Kara I (2001). The effects of different 4-aminopyridine and morphine combinations on the intensity of morphine abstinence. Pharmacol Res.

[16] Alaei H, Moloudi R, Sarkaki A, Azizi-Malekabadi H, Hanninen O (2007). Daily running promotes spatial learning and memory in rats. J Sports Sci Med.

[17] Mathes WF, Kanarek RB (2006). Chronic running wheel activity attenuates the antinociceptive actions of morphine and morphine-6-glucoronide administration into the periaqueductal gray in rats. Pharmacol Biochem Behav.

[18] Haghparast A, Esmaeili A (2007). Effects of morphine and lidocaine administration into the cuneiformis nucleus of rats on acute and chronic pain modulation by formalin test. J Birjand Univ Med Sci.

[19] Haghparast A, Alizadeh A, Motamedi F Effect of subcutaneous morphine injection on neuronal activity in the nucleus cuneiformis of rat.

[20] Hoveida R, Alaei H, Oryana S, Parivara K, Reisi P (2011). Treadmill running improves spatial memory in an animal model of Alzheimer’s disease.

[21] Lett BT, Grant VL, Koh MT, Flynn G (2001). Prior experience with wheel running produces cross-tolerance to the rewarding effect of morphine. Pharmacol. Biochem Behav.

[22] Govern Mc MK (2005). The effect of exercise on the brain. Biology.

[23] Aiaei H, Esmaeili M, Nasimi A (2005). Pourshanazari A. Ascorbic acid decreases morphine self-administration and withdrawal symptom in rats. Pathophysiology.

[24] Pourshanazari AA, Alaei H, Rafati A (2000). Effect of electrical stimulation of nucleus raphe dorsalis on initiation of morphine self-administration in rats. Med J Islamic Acad Sci.

[25] Esch T, Stefano GB The neurobiology of pleasure,rewaed processes, addiction and their health implication. Neuro Endocrinol Lett.

[26] Cereb J, Ouchi Y, Yoshikawa E, Futatsubashi M, Okada H, Torizuka T (2002). Effect of simple motor performance on regional dopamine release in the striatum in Parkinson disease patients and healthy subjects:a positron emission tomography study. Blood Flow Metab.

[27] Fisher BE, Petzinger GM, Nixon K, Hogg E, Bremmer S, Meshul CK (2004). Exercise-induced behavioral recovery and neuroplasticity in the 1-methyl-4-phenyl-1, 2, 3, 6-tetrahydropyridine-lesioned mouse basal ganglia;. J Neurosci Res.

[28] Tzchentke TM, 2000, Department of pharmacology Research and Development, Grünenthal Gmbh, Aachen, Federal Republic of Germany The medial prefrontal cortex as a part of the brain reward system. Review Article Amine Acids.

[29] Alaeia H, Moloudia R, Sarkaki A (2008). Effects of treadmill running on mid-term memory and swim speed in the rat with Morris water maze test.

[30] Ahmadiasl N, Alaei H, Hänninen O (2003). Effect of exercise on learning, memory and levels of 2. epinephrine in rats’ hippocampus. J Sports Sci Med.

[31] Uysal N, Tugyan K, Kayatekin BM, Acikgoz O, Bagriyanik HA, Gonenc S (2005). The effects of regular aerobic exersice in adolescent period on hippocampal neuron density-apoptosis and spatial memory. Neurosci lett.

